# Impact of Fresh Leaf Elements on Flavor Components and Aroma Quality in Ancient Dancong Tea Gardens Across Varying Altitudes

**DOI:** 10.3390/plants14091339

**Published:** 2025-04-29

**Authors:** Xinyuan Lin, Wei Huang, Zihao Qiu, Jiyuan Yao, Hongbo Zhao, Waqar Khan, Binmei Sun, Shaoqun Liu, Peng Zheng

**Affiliations:** College of Horticulture, South China Agricultural University, Guangzhou 510642, China; imxyuanlin@stu.scau.edu.cn (X.L.); huangwei_chris@163.com (W.H.); scau20222018004@stu.scau.edu.cn (Z.Q.); yaojuan@stu.scau.edu.cn (J.Y.); rhbzhao@163.com (H.Z.); waqar.khan399@scau.edu.cn (W.K.); binmei@scau.edu.cn (B.S.)

**Keywords:** Dancong tea, ancient tea garden, flavor component, aroma compound, element

## Abstract

China′s ancient tea gardens boast a rich resource, and their unique environmental conditions cause their quality differences. In this study, flavor components and aroma compounds of fresh leaves from four Dancong ancient tea gardens (Zimao (ZMF), Baixiang (BX), Xialiao (HSK), and Da′an (DAF)) at different altitudes were determined by HPLC and GC–MS and then correlated with elemental contents. The results showed that low-altitude tea gardens ZMF had a higher caffeine content (5.21%) and astringency index (0.82) compared to other high-altitude tea gardens, which led to a more bitter taste and astringent sensation. Fresh leaves from ZMF had a higher content of linalool (151.31 μg/kg) and geraniol (61.09 μg/kg) than those of other tea gardens. Correlation analysis showed that the bitter and astringent indexes had a strong correlation with element N content (correlation coefficient: 0.74, 0.48); volatile compounds had significant positive or negative correlations with various elemental contents, among which the correlation coefficient between element Al content and linalool content of fresh leaves was −0.83 (*p* < 0.001). The fresh leaves of ZMF tea gardens had a higher N content and lower Al, Si, and Hf content, which may cause more bitterness astringency and differences in volatile compounds in their teas than those of higher altitude tea gardens. The results of the study further provide guidance for the scientific management of Dancong ancient tea gardens.

## 1. Introduction

Tea has a range of secondary metabolites, including tea polyphenols, amino acids, soluble sugars, alkaloids, and terpenes. These components are closely related to the quality of tea and contribute to its diverse taste, flavor, and health benefits [1]. Catechins are the most important secondary metabolites in tea and have a variety of physiological effects (e.g., antioxidant and anticancer), and they have also been associated with lower lipid levels and weight loss [2]. About 70% of tea polyphenols are catechins, which give tea its characteristic bitter flavor [3]. Ancient tea trees are defined as wild ancient tea trees and their communities in natural forests, semi-domesticated wild tea trees, or tea trees in ancient tea gardens cultivated for more than 100 years [4]. Tea leaves collected and processed from ancient tea gardens have been widely recognized as having better quality and preservation value due to lower yields, more nutrients absorbed by a well-developed root system, and more significant changes in chemical composition [5,6]. Ancient tea trees are rich in genetic diversity, contain unique tea functional components, and have favorable advantages regarding resilience, yield, and biochemistry-related traits [7]. As previously indicated, a significant proportion of tea polyphenols, total catechins, free amino acids, gallic acid, soluble sugars, and caffeine were found within the fresh tea leaves from ancient tea gardens. However, the polyphenol/amino acid ratio and the ester catechin content were notably lower, particularly that of epigallocatechin gallate (EGCG) [8]. Pu-erh tea from ancient tea gardens has been reported to have high levels of free amino acids, fatty acids, phenolic acids, nucleosides, and nucleobases, but low levels of flavonoids and caffeine congeners [9]. The ancient tea tree resources of Fenghuang Mountain in Chaozhou City, Guangdong Province have been recognized as the world′s largest collection of rare, multi-fragrance, multi-species, cultivated rare tea tree resources. Fenghuang Mountain Ancient Tea Garden is one of China′s unique cultivated ancient tea tree communities, and Fenghuang Dancong tea has a history of more than 900 years [10]. The altitude of Fenghuang Mountain in Chaozhou ranges from 350 m to 1498 m above sea level, with an average annual temperature of 20 °C and an average annual rainfall of 2160 mm. While many excellent Dancong ancient tea trees have disappeared due to natural aging and natural disasters, some have been sharply reduced due to environmental factors such as excessive harvesting, man-made tree felling, or deterioration of the ecological environment. Due to the negligent management and protection of ancient tea trees, the destruction of ancient tea tree resources continues to intensify, the ancient tea tree germplasm resource base is on the verge of a serious crisis, and the death of ancient tea trees is on the rise. Scientifically grasping the contradiction between the growth and production of Dancong ancient tea trees is important to maintain the value of Dancong tea trees after restoration.

Volatile compounds in fresh tea leaves form the basis for the production of tea aroma. The composition of volatile compounds in fresh tea leaves is influenced by the cultivation environment, including factors such as air quality, rainfall, light, temperature, humidity, altitude, and soil conditions [11]. The types of volatile compounds and their contents are greater in high-altitude tea, and some of the odors of fresh tea leaves, such as grassy, earthy, or hay-like odors, are mainly found in relatively low-altitude tea gardens, whereas fresh tea leaves from higher altitudes are imbued with more sweet, floral, and honeyed flavors [12]. A study comparing fresh leaves of Tieguanyin tea at different altitudes found that volatile aromatic compounds such as benzyl alcohol, phenylethanol, and acetophenone were found to be higher in tea leaves at higher altitudes [13]. Tea tree varieties suitable for making oolong tea show a highly expanded family of terpene synthase genes, and their main aroma compounds are terpene aroma substances including nerolidol, geraniol, and α-farnesene [14,15,16].

Mineral elements in tea have been shown to affect the growth and quality of the tea plant [6]. Appropriate application of nitrogen fertilizer can balance the lipid metabolism of tea and the formation of aroma source compounds, which can help improve the quality of tea. However, excessive application of nitrogen may lead to aggravation of the grassy flavor of tea leaves, thus reducing the aroma quality [17]. Long-term nitrogen application significantly reduces the content of benzyl alcohol and phenylethanol in fresh tea leaves, as well as the content of *trans*-nerolidol and indole during withering, which is detrimental to the formation of floral and fruity aroma complexes [18]. The application of organic fertilizers significantly increases the concentration of green tea aroma compounds such as D-limonene, *cis*-jasmone, nonanal, linalool, linalool hexanoate, and *cis*-3-hexenol benzoate [18]. Magnesium treatment is detrimental to the synthesis of tea quality-related secondary metabolites and amino acids, and the contents of tea polyphenols, caffeine, flavonoids, aqueous extracts, and free amino acids showed a decreasing trend with Mg concentration [19]. It has been shown that the concentrations of caffeine and free amino acids in tea leaves treated with different concentrations of Pb significantly decreased, but the concentration of catechins increased with the increase in Pb concentration [20]. It was found that Cu and P elements in young and mature leaves of tea plants had a significant effect on the content of EGCG and tea polyphenols [21]. In addition, L-theanine content was reduced due to decreased synthesis and increased degradation as a result of nutritional deficiencies [22]. Soil nutrient deficiencies reduce the abundance of many aromatic compounds (linalool and its oxides and methyl salicylate, etc.), reducing tea quality and aroma [23].

A study on the correlation between the quality of tea leaves from Dancong ancient tea gardens and the elements contained therein has not yet been reported. In our study, we analyzed the concentrations of all the elements in the fresh leaves of four different Dancong ancient tea gardens, combined with the determination of tea taste components, aroma compounds, and other indexes, and carried out multivariate statistical analysis to screen the main influencing factors affecting the quality of the tea leaves of the four different Dancong ancient tea gardens and analyzed their correlation. This study focuses on the differences in elemental content, flavor compounds, and aroma quality of fresh leaves from Dancong ancient tea gardens across different altitudes. Additionally, it explores the factors affecting the differences in the quality of fresh leaves of ancient tree tea at different altitudes from the perspective of elemental content differences. This study is important in theory and practice to improve the economic benefits of Dancong ancient tea trees and improve the quality of tea, and at the same time, it provides a theoretical basis for the scientific management of ancient tea gardens.

## 2. Results

### 2.1. Analysis of the Differences in Flavor Components of Fresh Leaves from Different Altitudes Dancong Ancient Tea Gardens

#### 2.1.1. ZMF Fresh Leaves from Low-Altitude Dancong Ancient Tea Gardens Have a More Bitter Flavor

**Figure 1:**
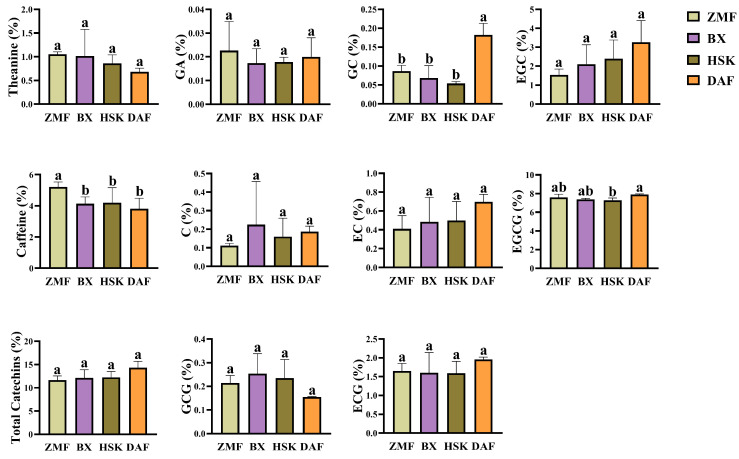
Content analysis of theanine, caffeine, and catechins in the leaves of four different Dancong ancient tea gardens (ZMF, BX, HSK, and DAF). Histogram and error bars represent mean ± standard deviation (SD) (%), and different letters (a, b) indicate the differences according to the Tukey HSD comparison method (*p* < 0.05), *n* = 3.

Theanine, catechin, and caffeine are essential inclusions in tea that provide flavor and function. In terms of total theanine, the highest content (1.05%) was found in Zimao (ZMF) at the lowest altitude (578.13 m). Figure 1 shows the analysis of the content of theanine, caffeine and catechins in leaves of four different Dancong ancient tea gardens. In contrast, the theanine content decreased with altitude in ancient tea gardens at higher altitudes (965.91 m~1131.63 m): the Baixiang (BX) theanine content was 1.02%, while the Da′an (DAF) theanine content was the lowest (0.68%). For caffeine, the content of caffeine in ZMF tea was significantly higher than that in other tea gardens (5.21%) and 25.95% higher than that in the BX tea garden with the highest altitude; the difference in caffeine content among BX, Xialiao (HSK), and DAF tea was not significant. For total catechins, the highest content was found in DAF tea (14.36%) and the lowest in ZMF (11.64%). Among the indicators of catechin fractions, EGCG had the highest content, followed by EGC and ECG content, and then EC, GCG, and C content, and GC and GA had the lowest content. In terms of catechin monomers, the GC and EGCG contents of tea from DAF at 965.13 m above sea level were higher than the contents of these two in other ancient tea plantation teas, which were 0.18% and 7.90%, respectively. The content of GC and EGCG in ZMF tea at lower altitudes was second only to DAF, with 0.09% and 7.61%, respectively. In terms of EGC content, the three tea gardens at higher altitude showed a pattern of decreasing with increasing altitude at 2.10% (BX), 2.39% (HSK), and 3.27% (DAF), while the ZMF content at lower altitude was 1.54%. The content of ester catechins (EGCG, ECG) in different Dancong ancient tea gardens had the same pattern, in which the ECG content of ZMF was the same as that of EGCG, only second to that of DAF, which was 1.65%. During processing, catechins undergo chemical reactions such as decomposition and conversion. Regarding total catechins, DAF had the highest content, and ZMF had the lowest. This suggests that fresh leaves with DAF are more resistant to processing and have a greater potential for quality preservation and enhancement after processing.

The astringency of tea is mainly due to the catechins in polyphenols, especially ester catechins. Therefore, we use the proportion of ester catechins to the total catechin content to assess the astringency of tea, which is defined as the astringency index. It was found that the astringency index of tea was mainly contributed by epigallocatechin gallate (EGCG) and epigallocatechin gallate (ECG), while gallocatechin gallate (GCG) had less effect on the astringency index. In contrast, ZMF, with the lowest elevation, had the highest total astringency index (0.82), and DAF had the lowest total astringency index (0.70) (Table 1). Taken together, the results show that tea from the ZMF Dancong ancient tea garden with the lowest altitude is more bitter and astringent than that of other Dancong ancient tea gardens due to it having a higher caffeine content and higher astringency index than other tea gardens at higher altitudes. The DAF Dancong ancient tea garden had the lowest caffeine content and astringency index, and its teas may be less bitter and astringent. In addition, tea gardens at higher altitudes have less caffeine than tea gardens at lower altitudes. The highest altitude BX tea gardens may have a more neutral flavor compared to other tea gardens due to their higher theanine and lower caffeine content.

#### 2.1.2. Principal Component Analysis of Non-Volatile Substances in Fresh Leaves of Different Ancient Tea Gardens

**Figure 2:**
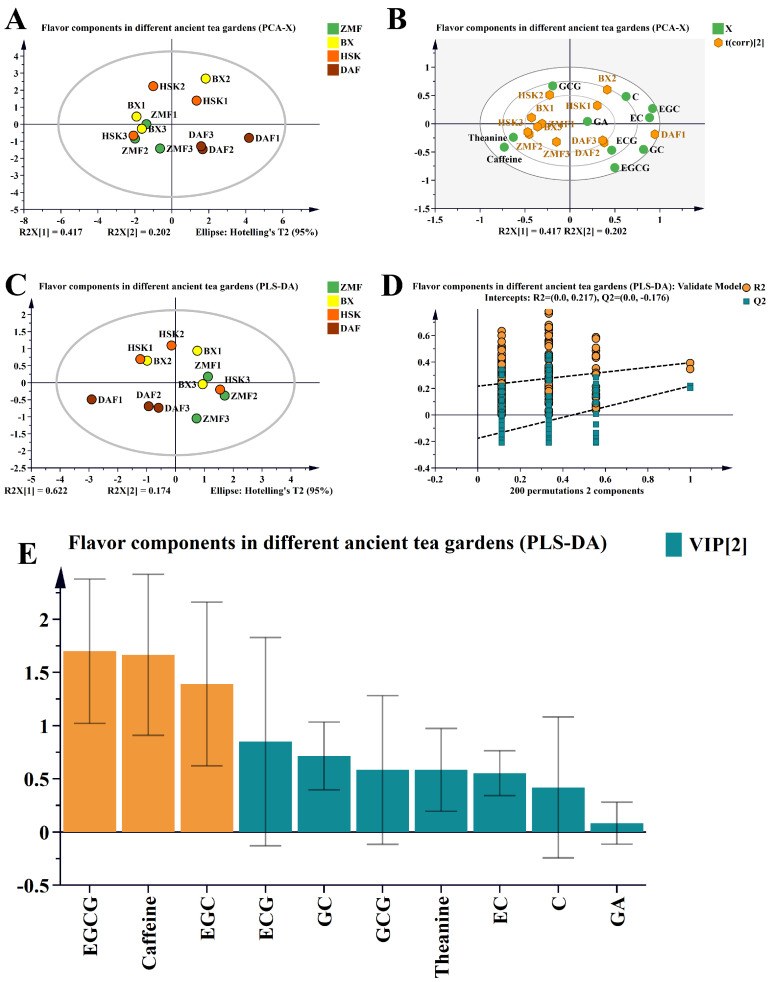
Multivariate statistical analysis of non-volatile compounds identified in fresh leaves of four different Dancong ancient tea gardens. (**A**) PCA plots of non-volatile compounds in fresh leaves of different Dancong ancient tea gardens; (**B**) double-label plots of non-volatile compounds in fresh leaves of different Dancong ancient tea gardens; (**C**) PLS-DA score plots of non-volatile compounds in fresh leaves of different Dancong ancient tea gardens. (**D**) Cross-validation results: the intercepts of the Q2 replica lines of the cross-validated model for the 200 comparisons were less than 0, which indicated that the PLS-DA discrimination model had not been overfitted and that the model is relatively reliable. (**E**) VIP score plot: orange color bar indicates non-volatile compounds with VIP > 1; green color indicates non-volatile compounds with VIP < 1.

We used multivariate statistical PCA and PLS-DA analysis methods to analyze the non-volatile compounds of different ancient tea gardens obtained, as described below (Figure 2). The results showed that there were some differences in the non-volatile compounds in the fresh leaves of different ancient tea gardens. The double-labeled plot (Figure 2B) showed that the levels of ECG, EGCG, and GC were higher in DAF, while theanine and caffeine were higher in ZMF.

Based on the above results, we built a PLS-DA model (Figure 2C–E), which distinguished the non-volatile compounds characterizing different ancient tea gardens. This constructed model was predictive without overfitting (R2 > Q2 and Q2 intercept < 0, Figure 2D). Based on the above results, we obtained the VIP values of each non-volatile compound and screened the variables with VIP > 1 (Figure 2E), such as EGCG, caffeine, and EGC, suggesting that these compounds are important in the differentiation of non-volatile components associated with tea quality of different ancient tea gardens and may be the key factors for the differences in the taste of the four different Dancong ancient tea gardens.

### 2.2. Differential Analysis of Aroma Compounds of Fresh Leaves of Different Altitudes Dancong Ancient Tea Gardens

#### 2.2.1. Low-Altitude ZMF Fresh Leave Have Highest Concentration Yet Least Variety of Aroma Compounds

**Figure 3:**
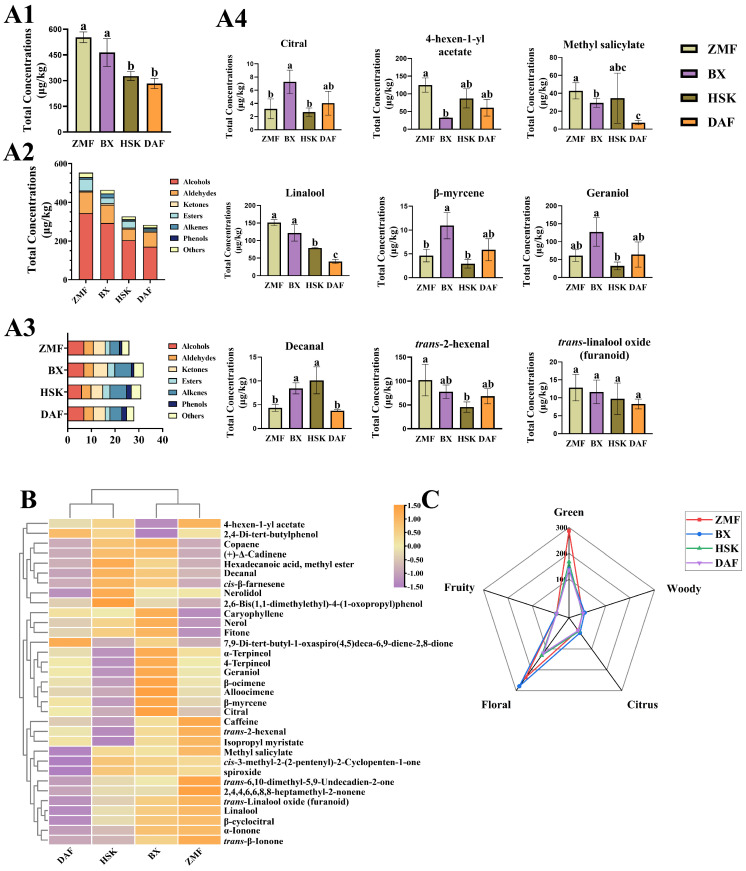
The differences in volatile compounds content in fresh leaves of four different Dancong ancient tea gardens were analyzed from different perspectives. (**A1**) represents the differences in the total concentration of volatile substances in fresh leaves of four different Dancong ancient tea gardens; (**A2**) represents the concentration of different categories of volatile substances identified; (**A3**) represents the number of different categories of volatile substances identified; (**A4**) represents the differences in the concentration of the nine volatile substances with the highest concentration identified; (**B**) represents a heat map of the concentration of identified volatiles; (**C**) represents the radar plot of total amount of aroma compounds of different types of fresh leaves from different Dancong ancient tea gardens. Histogram and error bars represent mean ± standard deviation (SD) (%), and different letters (a, b, c) indicate the differences according to the Tukey HSD comparison method (*p* < 0.05), *n* = 3.

After headspace solid-phase microextraction (SPME), the volatiles of tea leaves were separated and identified using gas chromatography–mass spectrometry (GC–MS). After the removal of disturbing volatile component impurities, 32 volatile compounds were detected in BX, 28 volatile compounds were detected in DAF, 31 volatile compounds were detected in HSK, and 26 volatile compounds were detected in ZMF (Figure 3C). Among the identified compounds seven alcohols, four aldehydes, six ketones, three esters, seven alkenes, two phenols, and three other volatile compounds were included. We identified the same 23 volatile compounds in the fresh leaves of the four different Dancong ancient tea gardens.

Overall, the total volatile compounds were 552.65 μg/kg in the ZMF tea garden, 463.79 μg/kg in BX, 326.86 μg/kg in HSK, and 283.50 μg/kg in the DAF tea garden. The total volatile compounds were highest in the ZMF tea garden at the lower elevation and lowest in the DAF tea garden; and values for both the ZMF and BX tea gardens were significantly higher than for the DAF and HSK tea gardens (*p* < 0.05). The total volatile compounds of both ZMF and BX tea gardens were considerably higher than those of DAF and HSK tea gardens. Among them, the total volatile substance values of the ZMF tea garden were not significant compared to those of the BX tea garden, which were 19.16% higher than those of the BX tea garden (Figure 3(A1–A4)). The results indicated that the fresh leaves of the lower altitude ZMF Dancong ancient tea garden had similar fresh leaf volatile compound concentrations as those of the high-altitude tea garden BX and were significantly higher than those of the other two high-altitude tea gardens (*p* < 0.05) (DAF, BX).

The volatile fractions with high content in fresh leaves were alcohols and esters, followed by aldehydes and olefins, with ketones and phenolics at very low levels. Among the alcohols detected, the substances with higher contents in fresh leaves were linalool, geraniol, and 4-hexen-1-yl acetate, while among the aldehydes and esters detected, the substances with the highest contents in fresh leaves were *trans*-2-hexenal and methyl salicylate, respectively. Among them, the ZMF tea garden had the most alcohols, aldehydes, and esters, which were 343.82 μg/kg, 110.89 μg/kg, and 59.79 μg/kg, respectively; alcohols were 17.32%, 68.44%, and 101.21% higher than those of BX, HSK, and DAF tea gardens, respectively; aldehydes were 17.25%, 87.27% and 44.42%, respectively; and esters were 99.60%, 70.37%, and 678.84% higher than those of BX, HSK, and DAF tea gardens, respectively. BX had the most ketones and olefins, with 7.23 μg/kg and 20.00 μg/kg, respectively (Figure 3B). We summarized the different aroma types of the four different Dancong ancient tea gardens based on the aroma characteristics of the identified volatiles (Table S4). From the radar chart, we can find that the fresh leaves of the ZMF tea garden had the highest content of green aroma (286.84 μg/kg) compared to the other Dancong ancient tea gardens; the DAF tea garden had the lowest content of green aroma volatiles, which was 52.34% less than the content of the ZMF. While the fresh leaves of the BX tea garden had the highest content of floral aroma (278.88 μg/kg), the content of this aroma type in the ZMF tea garden was 15.25% lower than that of BX.

We selected the nine most abundant volatile compounds identified for analysis in order to visually observe the differences of different volatile compounds among different ancient tea gardens, as shown in Figure 3. It was found that the contents of methyl salicylate and linalool in the fresh leaf samples from the four ancient tea gardens showed similar differences to the total amount, with the highest levels in ZMF (59.26 μg/kg, 151.31 μg/kg) and BX (29.33 μg/kg, 121.98 μg/kg). The content of citral in BX was significantly higher than that in ZMF and HSK ancient tea gardens (7.27 μg/kg); β-myrcene had the highest level (10.94 μg/kg) and was significant to HSK and ZMF; geraniol had the highest level and was significant to DAF; and in ZMF the level of 4-hexen-1-yl acetate was the highest and significant relative to BX; *trans*-2-hexenal was the highest and significant relative to HSK (*p* < 0.05). Meanwhile, HSK and BX had higher decanal content than ZMF and DAF. A clustered heat map of the 33 volatile compounds identified their content components. The heat map showed approximately the same pattern as the above results. Among them, the volatile content of the BX ancient tea garden was high compared to that of other tea plantations, as shown by the fact that α-terpineol, terpinen-4-ol, geraniol, β-ocimene, alloocimene, β-myrcene, citral, caryophyllene, and nerol values were higher than those of the other three ancient tea gardens. From the cluster analysis, the accumulation pattern of volatile compounds in the lowest altitude tea garden ZMF was similar to that of the highest altitude tea garden, BX. The volatile compound accumulation pattern of the HSK tea garden was similar to that of DAF tea garden. In summary, the results showed that the lower altitude ZMF tea garden had similar fresh leaf volatile matter content to the highest altitude BX tea garden and had higher fresh leaf volatile matter content compared to the other two high-altitude tea gardens (DAF and HSK).

#### 2.2.2. Multivariate Statistical Analysis of Volatile Compounds in Tea Fresh Leaves

**Figure 4:**
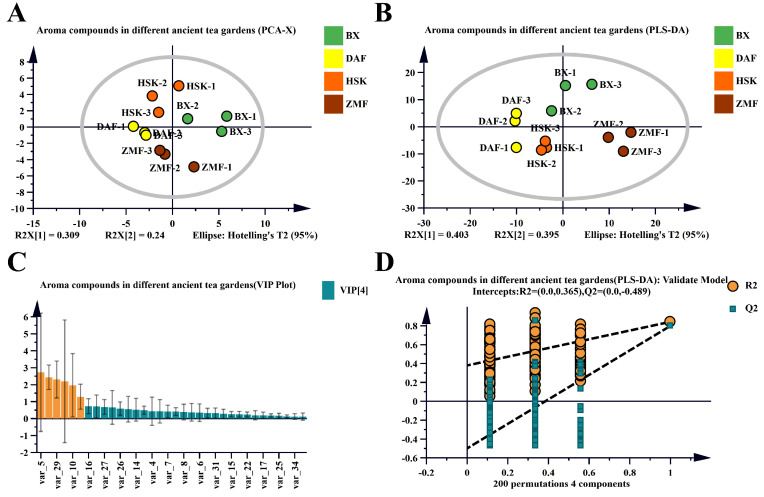
Multivariate statistical analysis of volatile compounds identified in fresh leaves of four different Dancong ancient tea gardens. (**A**) PCA plots of volatile compounds in fresh leaves of different Dancong ancient tea gardens; (**B**) PLS-DA score plots of volatile compounds in fresh leaves of different Dancong ancient tea gardens; (**C**) VIP scoring plots: orange color bars indicate volatile compounds with VIP > 1; green color indicates volatile compounds with VIP < 1. (**D**) Cross-validation results: 200 comparisons of the cross-validated model, the intercept of the Q2 replica line is less than 0, indicating that the PLS-DA discriminant model is not overfitted and the model is relatively reliable.

Based on the 33 volatile compounds previously screened in the fresh leaf species of four different altitude Dancong ancient tea gardens, the differences and similarities among the samples were assessed using principal component analysis (PCA). The PCA model well separated the two principal components, explaining 54.9% of the total variance (Figure 4A). The results showed that the differences in the volatile compounds contained in the fresh leaves of the four different ancient tea gardens were significantly differentiated, while BX, HSK, and DAF in three of the higher altitude tea gardens were relatively close to each other in the figure.

To further distinguish the mentioned volatile compounds in fresh leaves of four different ancient tea gardens, to screen out the potential key volatile compounds contributing to the differences in tea aroma among the four different ancient tea gardens, we used partial least squares-discriminant analysis (PLS-DA) to determine the variable importance in the projected values (VIP). Figure 4B–D demonstrate the PLS-DA models constructed based on the volatile compounds of fresh leaves from different ancient tea plantations; the constructed models were suitably predictable by 200 permutation tests (R2 = 0.365, Q2 = −0.489), and there was no overfitting, suggesting that the models were reliable (Figure 4B). Screening of substances with VIP > 1 (orange part of Figure 4C) resulted in six volatile compounds, including three alcohols, two aldehydes, and one ester. These six substances were linalool, *trans*-2-hexenal, 4-hexen-1-yl acetate, geraniol, methyl salicylate, and decanal in descending order of VIP value.

The contribution of a volatile substance to the overall aroma is related to its concentration and odor activity value (OAV), which refers to the minimum concentration at which a compound has a perceptible odor. Volatile components that satisfy the conditions of VIP > 1 and OAV ≥ 1 and whose aroma has been identified were selected as potential key aroma substances. Based on this discrimination, we further identified six volatile compounds as potential key aroma substances: three green aroma related compounds, two floral aroma related compounds, and one citrus aroma related compound. Among them, linalool had the highest VIP value (2.73), with OAV ranging from 184.14 to 687.78, which was inferred to be the major contributor to the aroma (Table 2). Geraniol also had high OAV values (29.43 to 115.72) in the four tea gardens. In summary, the results showed that the fresh leaves from the low-altitude ZMF Dancong tea garden had a more significant floral and green aroma compared to the fresh leaves from the other three high-altitude Dancong tea gardens, BX, HSK, and DAF.

### 2.3. Analysis of Elemental Content of Fresh Leaves in Different Ancient Tea Gardens

#### 2.3.1. Differences in Elemental Content of Fresh Leaves in Different Ancient Tea Gardens

**Figure 5:**
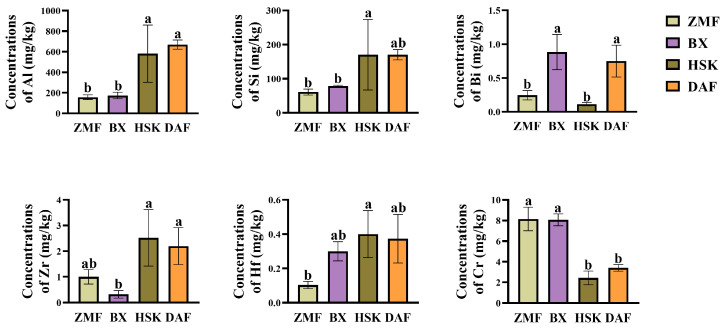
Content analysis of elemental contents with significant differences in the leaves of four different Dancong ancient tea gardens. Histogram and error bars represent mean ± standard deviation (SD) (%), and different letters (a, b) indicate the differences according to the Tukey HSD comparison method (*p* < 0.05), *n* = 3.

As the saying goes, “Good tea comes out of high mountain clouds and mist”. The low-altitude Dancong ancient tea garden ZMF selected in this study has a higher content of aroma than other high-altitude tea gardens, even though it is slightly inferior to other high-altitude tea gardens in terms of flavor compounds. In addition, in the three higher altitude tea gardens (BX, HSK, DAF), there were some significant differences in the aroma compounds of their fresh leaves. In order to further investigate the reasons for these phenomena and differences, we focused on the elemental contents of the fresh leaves of these four different Dancong ancient tea gardens. Tea fresh leaf samples were collected from the corresponding loci of the studied tea garden soils, which were digested and determined by full scanning with ICP-MS, and the elemental contents of the tea fresh leaves are shown in Table S1. There were differences in the contents of different elements in the four ancient tea gardens, and the average size of the elemental contents of the tea fresh leaves were in the following order: N > K > P > Mg > Ca > Al > Mn > Na > Fe > Si > Zn > Sr > Li > Ba > B > Cu > Cr > Ni > Ti > Zr > As > Bi > Co > Mo > Hf > V > Sn. Among all the detected elements, six elements (Al, Si, Bi, Zr, Hf, and Cr) significantly differed in the fresh leaves of four different Dancong ancient tea gardens (Figure 5). The elemental contents of Al, Si, Zr, and Hf showed similar trends of differences among the four tea gardens, with the Al content in fresh leaves of the lower altitude ZMF tea garden being 73.09% and 76.64% lower than that in fresh leaves of the HSK and DAF tea gardens, respectively, whereas there was no significant difference in the Al content of fresh leaves of the two tea gardens compared to that of the highest altitude BX tea garden, which was only 9.9% lower than that of BX (*p* < 0.05). For Cr content, the content of this element in fresh leaves of ZMF tea garden at a lower altitude was not significantly different from that in fresh leaves of the BX tea garden at the highest altitude, which was 0.99% higher than that of BX (*p* < 0.05); however, it was significantly higher than that of HSK and DAF tea gardens, which were 234.79% and 138.67% higher, respectively (*p* < 0.05). Therefore, it was presumed that the differences in elemental content of fresh leaves at different altitudes may be responsible for the differences in the content of volatile and non-volatile substances among different tea gardens. The differences in these compounds, in turn, affect the quality of the tea.

#### 2.3.2. Principal Component Analysis of Fresh Leaf Elements in Different Ancient Tea Gardens

In order to screen out the elements that contributed most to the quality of the fresh leaves of the four different old ancient gardens, we constructed PCA and PLS-DA models by performing multivariate statistical analysis on the above-detected indicators of the whole element contents (Figure S1). The PCA model demonstrated the separation of various elements in the fresh leaves of four different ancient tea gardens, in which the elemental contents of DAF and HSK were close to each other. At the same time, there was a large separation from BX and ZMF (Figure S1A), indicating that the elemental contents of the fresh leaves of the latter two ancient tea gardens differed from those of the former two. It is hypothesized that the differences in tea quality among these four different tea gardens may be correlated with the differences in the content of volatile and non-volatile compounds due to their fresh leaf elements. A PLS-DA model was constructed, which explained 79.2% of the total variance (Figure S1B), and it passed the test, proving the model′s reliability (R2 = 0.235, Q2 = −0.131, Figure S1C). We used the model to obtain the elements with VIP values > 1, which were, in order of magnitude, according to the VIP values, N, Ca, Al, Mg, Na, K, and P. It was shown that these elements were the main reason for the differentiation of the differences in elemental contents in fresh leaves from four different ancient tea gardens (Figure S1D).

### 2.4. Correlation Analysis of Fresh Leaf Elements with Key Flavor Compounds and Potential Key Aroma Substances in Different Ancient Tea Gardens

In order to further study the relationship between fresh leaf elements and tea quality differences in four different Dancong ancient tea gardens, we chose the above-screened key elements with significant differences (*p* < 0.05) and based on the content of the above-measured elements (N, Ca, Al, Mg, Na, K, P, Si, Bi, Zr, Hf, Cr). Then, we carried out correlation analyses with the above identified key flavor compounds and key aroma compounds, respectively, to speculate on the key reasons for the quality differences (Figure 7).

#### 2.4.1. Heat Map of Correlation Between Fresh Leaf Elements and Key Flavor Compounds in Different Ancient Tea Gardens

**Figure 6:**
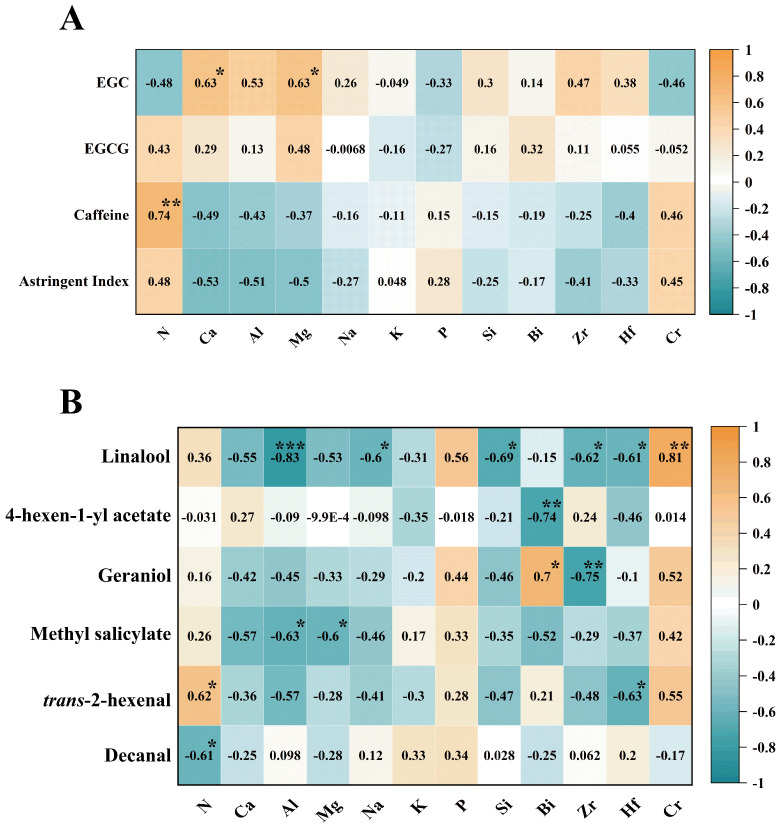
(**A**) Correlation heat map between the concentrations of 12 elements affecting the differences in fresh leaves of four different Dancong ancient tea gardens and the concentrations of key inclusions and quality indexes therein. (**B**) Correlation heat map between the concentrations of 12 elements affecting the differences in fresh leaves of four different Dancong ancient tea gardens and the concentrations of six potentially key aroma compounds. The darker/lighter color of the squares represents higher/lower correlation, orange indicates a positive correlation, green indicates a negative correlation, and the numbers in the squares indicate the Pearson correlation coefficients, and “*” indicates the significance of this correlation at *p* < 0.05, and “**” indicates the significance of the correlation at *p* < 0.01, and “***” indicates the significance of the correlation at *p* < 0.001.

We conducted a correlation analysis (Figure 6A) using the above-obtained contents of all elements in the fresh leaves of four different ancient tea gardens species with the screened key inclusions composition and content. As shown in Figure 6A, elemental N and caffeine contents in fresh leaves of the four ancient tea gardens were highly significantly correlated, with a correlation coefficient of 0.74. This suggests that nitrogen in fresh leaves has a high correlation with tea caffeine content, which may further cause changes in tea flavor. In addition, Ca and Mg elements in the fresh leaves of different ancient tea gardens were significantly and positively correlated with EGC in tea fresh leaves, with correlation coefficients of 0.63 in all cases. The elemental contents of Ca, Al, Mg, Na, K, Si, Bi, Zr, and Hf were negatively correlated with caffeine content in four different ancient tea gardens, and N and Cr had high negative correlation coefficients with EGC content, which were −0.48 and −0.46, respectively. In addition, N, K, and P were positively correlated with astringency indices (0.48, 0.048 and 0.28), whereas the other elements were negatively correlated with the astringency index.

The correlation analysis showed that the richness of N, Ca, and Mg elements in the fresh leaves of tea might be beneficial to the synthesis and accumulation of flavor constituents, and that the contents of these elements in the fresh leaves might be the reason for the differences in the flavor qualities of the four different Dancong ancient tea gardens. On the whole, the fresh leaves of the ZMF tea garden at lower altitudes had the highest N content, which might be the reason why its caffeine content was higher than that of the fresh leaves of the other three high-altitude Dancong ancient tea gardens.

#### 2.4.2. Heat Map of Correlation Between Fresh Leaf Elements and Key Aroma Compounds in Different Ancient Tea Gardens

Similarly, we correlated the above-screened potential key aroma substances with the key elements of the fresh leaves from four different ancient tea gardens to further explain the reasons for the differences in aroma quality of the tea leaves from the four different ancient tea gardens. As shown in Figure 6B, elemental N in fresh leaves was significantly correlated with *trans*-2-hexenal content, with a correlation coefficient of 0.62 (*p* < 0.05); and significantly negatively correlated with decanal content, with a correlation coefficient of −0.61 (*p* < 0.05). The contents of Al, Na, Si, Zr, and Hf in fresh leaves were all significantly negatively correlated with the linalool content (Na, Si, Zr, and Hf: *p* < 0.05; Al: *p* < 0.001), among which the correlation between Al and linalool was highly significant and negatively correlated, indicating that the high content of Al may be detrimental to the accumulation of linalool in fresh leaves. While Cr content was in contrast to the above elements, it was significantly and positively correlated with linalool content (0.81) (*p* < 0.01). In addition to this, Al and Mg were significantly and negatively correlated with methyl salicylate content in fresh leaves (*p* < 0.05), while P was positively correlated with linalool, geraniol, methyl salicylate, *trans*-2-hexenal, and decanal content in fresh leaves. In combination with the aroma characteristics of the key aroma substances, elemental N favors the formation of green aroma in fresh leaves, while high contents of Al, Mg, and Bi inhibit the green aroma; the accumulation of Bi and Zr may enhance the floral aroma of fresh leaves.

Taken together, there was no significant difference in linalool content in fresh leaves of the ZMF tea garden at low altitude and the BX tea garden at highest altitude, while it was significantly higher than that of the other two tea gardens, probably because the fresh leaves of ZMF and BX contained lower content of Al and higher content of Cr, and also the higher content of Na, Si, Zr, and Hf might have inhibited the accumulation of linalool in HSK and DAF. In addition, the fresh leaves of the ZMF tea garden at low elevation had a more pronounced green aroma than those of the other three tea gardens, probably due to the higher N content and lower Al, Mg, and Bi contents in their fresh leaves.

## 3. Discussion

### 3.1. Elements and Taste Compounds of Dancong Ancient Tea Gardens

ZMF tea garden fresh leaves had the highest content of caffeine and theanine, while DAF tea garden fresh leaves had the least content of both. DAF tea garden fresh leaves had the highest total catechin content, and ZMF tea garden fresh leaves had the lowest total catechin content. This indicates that fresh leaves of DAF are resistant to processing and have the highest potential for quality maintenance and improvement after processing. In our study, elemental N in fresh leaves was highly significantly correlated with theanine and caffeine content, and it has been confirmed that the content of caffeine [22] and theanine [28] in tea leaves is related to nitrogen metabolism, which is an important element for the synthesis of these substances, which is in agreement with the previous studies. The correlation analysis between elements and inclusions in fresh leaves of Dancong tea gardens showed that the high content of elements N, Ca, Mg, and P in fresh leaves of ancient tea gardens is favorable for the synthesis of inclusions in fresh leaves. It has been shown that the free amino acid, caffeine, GA, and EGC contents are significantly higher in ancient tea gardens compared with modern tea gardens [8]. The results were similar to those of this study, and based on the correlation analysis, it was speculated that this might be related to the content of elements such as N (theanine, caffeine) and Ca (GA, EGC). Combined with soil production, the higher content of available nitrogen (AN) and total nitrogen (TN) in soil was unfavorable to the synthesis of caffeine in fresh leaves; the higher content of available potassium (AK) in soil was favorable to the synthesis of theanine in fresh leaves. Catechins and caffeine are important tea flavor quality-related compounds, and soil nutrient deficiencies not only significantly reduced the caffeine content of fresh tea, but also led to a decrease in amino acid content and ultimately to a significant increase in phenol-ammonia ratio [23], which affects the organoleptic quality of tea leaves.

Our study focused on the determination of elemental content in fresh leaves of tea. The elements in fresh leaves, however, originate from the uptake, translocation, and assimilation of soil background elements, which in turn affect the taste and aroma quality of tea leaves. Organic matter is an important component of soil, which contains various nutrients needed for plant growth, is the energy source for the life activities of soil microorganisms, and has a profound impact on soil physical, chemical, and biological properties. The maintenance of soil organic matter is essential to ensure the productivity of the agro-ecosystem [29]. Long-term cultivation of tea favors the accumulation of soil organic carbon and total nitrogen [30]. The increase of soil organic matter and potassium is conducive to the improvement of soil microbial activity and enzyme activity, thus stimulating the tea to increase yield and improve tea quality [31]. Increased application of potash fertilizer can improve the content of amino acids and caffeine in tea. Therefore, the scientific management of Dancong ancient tea gardens should focus on soil potassium, phosphorus, magnesium and related trace elements, especially to strengthen the guidance of potash fertilizer application.

In our study, fresh leaves from Dancong ancient tea gardens were sampled across four distinct altitude gradients (Table 3, arranged in ascending order: ZMF < DAF < HSK < BX). However, the analysis revealed non-significant differences in the concentrations of most non-volatile compounds among these altitude groups, with only minor variations observed in specific constituents. Elevation within a specific range enhances the nitrogen metabolism of tea trees and facilitates the accumulation of amino acids, etc. [24]. In our study, except for ZMF at lower altitudes, fresh leaf theanine levels in the other three Dancong ancient tea gardens showed an altitude-dependent trend, similar to previous studies. In addition, the abundance of tea polyphenols increased with increasing altitude and began to decrease at altitudes above 800 m [32]. Interestingly, in our study, the catechins sampled beyond 900 m above sea level showed a similar trend (Figure 1). The reason for the effect on non-volatiles may be related to the different environment, which in turn affects the uptake of soil nutrients by the tea tree, an aspect that has not been addressed in the present study and therefore is not clear.

### 3.2. Elements and Aroma Compounds of Dancong Ancient Tea Gardens

The volatile compounds contained in the fresh leaves of the tea plant provide aroma, and different types and concentration levels of volatile compounds produce unique aroma profiles for the tea, which can be used to differentiate between different teas [33]. Linalool and its oxides and geraniol are considered to be the key aroma compounds of Dancong teas, and these volatiles present pleasant floral and woody aromas. They are found in high levels in fresh leaves. Methyl salicylate is the base aroma compound of a variety of teas [34]. *Trans*-2-hexenal and decanal are volatile substances common to all fresh leaves. Low-grade aliphatic aldehydes in tea are one of the main components of tea leaves that present fresh aroma; *trans*-2-hexenal is the main volatile component of tea fresh leaves, and its content is high, which provides Dancong tea fresh leaves with a good aroma.

In addition, the tea tree produces volatile compounds that increase its ability to withstand stress. Some studies have shown that cold-induced *cis*-3-hexenol improves the drought resistance of tea tree [35]; plant-derived S-linalool and β-ocimene attract parasitoid wasps and thereby suppress the tea tree birch brown planthopper, *Triticum aestivum* [36]; a study identified *trans*-nerolidol as a volatile signal involved in tea tree defense against the tea small green leafhopper and tea tree anthracnose (*C. fruiticola*) [37]. Geraniol has a significant inhibitory effect on pathogens and fungi and also improves the parasitization of natural enemies of pests [38]. Tea trees release volatile compounds such as *trans*-2-hexenal and methyl salicylate that repel tea aphids (*Toxoptera aurantii*) [39], reducing tea tree infestation by small green leafhopper pests [40]. The fresh leaves of Dancong ancient tea trees all have high levels of *trans*-2-hexenal and methyl salicylate, which may provide better insect resistance for the tea trees.

ZMF had the highest total amount of volatile compounds, while BX had the largest variety of volatiles. According to our study and analysis, geraniol has the highest contribution in BX tea and may be able to represent its aroma profile; decanal has the highest contribution in HSK tea and may be able to represent its aroma profile; while linalool, *trans*-2-hexenal, methyl salicylate, 4-hexen-1-yl acetate were recognized as their characteristic aromas because of their high contribution in ZMF. Based on the correlation analysis between the volatiles in fresh leaves and the elemental contents of fresh leaves from four different Dancong ancient tea gardens, it was shown that high N content in fresh leaves may cause a decrease in decanal, but may favor the synthesis and accumulation of *trans*-2-hexenal; and that high levels of P may be able to promote the accumulation of most of the volatile metabolites in fresh leaves. In contrast, high levels of Al, Mg, and Na in fresh leaves may be detrimental to the synthesis and accumulation of most volatile compounds such as methyl salicylate and linalool, whereas, in cultivated production, soil nutrient deficiencies reduced the content of tea aroma compounds, and linalool and its oxides (linalool oxides I and II), α-pinitol, geraniol, *trans*-nerolidol, and methyl salicylate were all significantly lower [23]. Potassium deficiency causes a decrease in the accumulation of alcohols, esters, aldehydes, and other aromatic compounds in tea leaves [41]. So, potassium-rich tea gardens or applying potassium fertilizer can improve the aroma quality of tea to a certain extent, as studies have shown that K can significantly improve the aroma of tea [42]. The application of 450 kg/ha of nitrogen fertilizer inhibited the synthesis of aroma compounds of Lingtou Dancong in summer, but its synthesis was enhanced by application of 300 kg/ha in autumn [3]. It has been pointed out that excessive application of nitrogen fertilizer adversely affects the biosynthetic pathways of aroma compounds, reducing the aroma quality of tea leaves [43]. How the elements contained in the soil are transported to the fresh tea leaves and how they affect the quality of the tea leaves is a topic that can be researched in the future.

**Figure 7:**
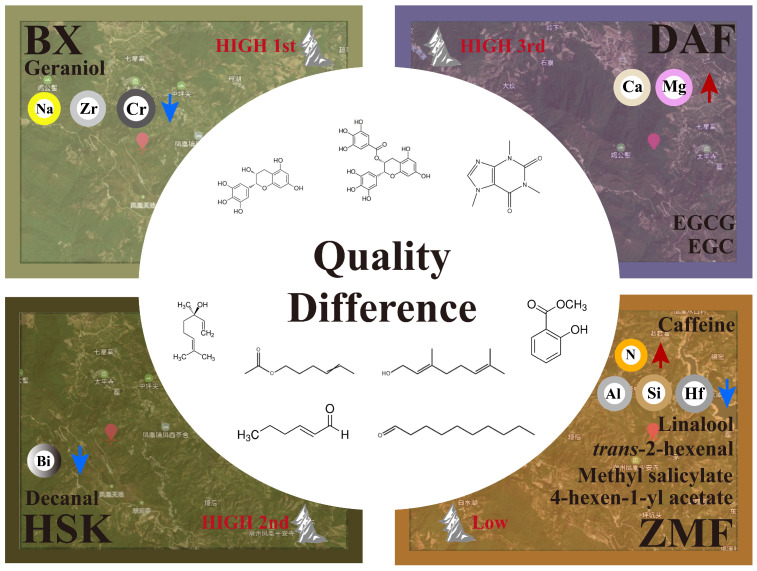
Schematic diagram of quality components and elements of different altitude Dancong ancient tea gardens. (Red upward and blue downward arrows indicate that the element has the highest content in this tea garden compared to other tea gardens, respectively).

In addition, it has been shown that altitude is negatively correlated with temperature and that cold stress induces the release of linalool and geraniol [44]. The linalool and geraniol contents of BX, HSK, and DAF in this study had a similar pattern to their study. However, the lower elevation ZMF had higher levels of both volatile compounds which, combined with the correlation analysis (Figure 6B), may be related to its higher N and P content and lower Al, which needs to be verified by investigating its soil background values (Figure 7).

## 4. Materials and Methods

### 4.1. Plant Materials

The fresh tea leaves were collected in April 2022. In four Dancong ancient tea gardens (Zimao (ZMF), Baixiang (BX), Xialiao (HSK), and Da′an (DAF), the geographical distribution is shown in Table 3) located in Fenghuang production area in Chaozhou City, Guangdong Province, ancient tea trees aged 100 years or more were selected, and those with uniform height, uniform canopy width, and no signs of pests and diseases were used in the experiment. The leaves of one bud and three leaves (refer to the position of the terminal bud) were plucked from the tea tree for the maturity of the clamped leaves, and approximately 250 g of samples were collected. The samples were put into polyethylene bags by removing the surface adherents, and all the samples were frozen in liquid nitrogen immediately, and then preserved in a refrigerator at −80 °C. The fresh leaf samples were rinsed with tap water and deionized water successively. A part of the fresh leaf samples was rinsed with tap water and deionized water successively, and the samples were put in a dry oven at 105 °C for 30 min after drying the water on the surface and then baked at 65 °C until a constant weight. Then, samples were ground through a 100-mesh nylon sieve and bagged. The other parts of the fresh leaf samples were lyophilized and powdered and stored in the refrigerator at −80 °C.

### 4.2. Determination of the Contents of Tea Flavor Compounds

Determination of catechins, caffeine, and theanine contents in tea samples by the method [3]. The contents of caffeine, theanine, and catechins were analyzed using HPLC (Waters Alliance 2695, 2489 UV/Vis; Waters Technologies, Milford, MA, USA) to compare retention times with standards. Concentrations of caffeine, theanine, and catechin were calculated using standard curves (Table S2). Acetonitrile and methanol (HPLC-grade) were purchased from Spectrum Chemical Manufacturing Co., Ltd. (Shanghai, China). Formic acid (Purity ≥ 99%) was purchased from Yuanye Bio-Technology Co., Ltd. (Shanghai, China).

For caffeine determination, 0.1 g of tea powder was mixed with 30 mL of 1.5% MgO solution and immersed in ultrapure water (*w*/*v*) at 100 °C. The resulting extract was double-filtered using a 0.22 μm microporous membrane, and 1 mL of the solution was collected. Then, 10 μL of the filtrate was injected into an XSelect HSS C18 SB column (4.6 × 250 mm, 5 μm) at a flow rate of 0.9 mL/min and a column temperature of 35 ± 1 °C. The mobile phase consisted of 100% methanol (A) and 100% ultrapure water (B) with 30% A/70% B isocratic elution. Caffeine was detected at 280 nm.

For the determination of theanine, 0.1 g of tea powder was immersed in 10 mL of ultrapure water at 100 °C. The supernatant was filtered using a 0.22 μm microporous membrane, and 10 μL of the filtrate was injected into an RP-C18 column (250 mm × 4.0 mm, 5 μm, 35 ± 1 °C). The flow rate was 0.5 mL/min. The HPLC program was based on the previous method [45]. Detection wavelength was set to 210 nm.

For the determination of catechins, 0.2 g of tea powder was extracted with 8 mL of 70% methanol in water. After filtering 1 mL of the supernatant through a 0.22 μm microporous membrane, it was injected into an XSelect HSS C18 SB column (4.6 × 250 mm, 5 μm). A gradient elution method was used, and the mobile phase consisted of 0.1% formic acid aqueous solution *v*/*v* (A) and 100% acetonitrile (B). The gradient elution started at 8% B for 5 min, increased to 25% from 5 to 14 min, and then decreased to 8% from 14 to 30 min. The detection wavelength was set to 280 nm. All experiments were conducted using independently prepared samples and in triplicate.

### 4.3. Determination of the Contents of Tea Volatiles Compounds

Identification and quantification of volatile substances refer to Chen et al. [46]. The headspace solid-phase microextraction (HS-SPME) method was applied to extract the volatile substances from tea. Specifically, 0.2 g tea powder, 5 mL of saturated sodium chloride solution, and 86.4 ng of ethyl decanoate were added into a headspace flask and sealed quickly. The divinylbenzene/carboxy/polydimethylsiloxane fibers (inner diameter 50/30 μm, length 2 cm) were preheated at 80 °C for 15 min and then inserted into the headspace flask and extracted for 40 min. After extraction, the SPME fibers were placed into the inlet of the gas chromatograph and resolved at 250 °C for 3 min. Three replicates were performed for each sample.

The GC–MS analysis was performed on an Agilent 7890B gas chromatograph and a 5977A mass spectrometer. An HP-5MS capillary column (30 m × 0.25 mm × 0.25 um film thickness) was used with high-purity helium as the carrier gas at a 1.0 mL/min flow rate in the non-shunt mode. The heating program was maintained at an initial temperature of 50 °C for 1 min, then increased to 220 °C at a rate of 5 °C/min and maintained for 5 min. The mass spectrometry ion source temperature was set at 230 °C, and the electron energy was set at 70 eV. The scanning range was from 30 to 400 atomic mass units (amu), and the solvent delay time was 4 min. Quantification of volatile compounds (μg/kg) was based on the peak areas of samples and comparison of the peak areas of ethyl decanoate [47].

### 4.4. Calculation of Tea Astringency Index

The proportion of ester catechin monomers to total catechins content was used to assess the astringency of tea, which is defined as the astringency index. The calculation formula is astringency index = Ca/Cc. Ca is the content of catechin monomer a(%), and Cc is the content of total catechins (%). The formulae are based on previous studies with modifications [48].

### 4.5. Calculation of Odor Activity Thresholds (OAVs)

OAV (odor activity threshold) is defined as the ratio of the concentration of an aroma compound in an aqueous solution to its odor threshold. The OAV method was used to evaluate each volatile compound′s contribution to the tea samples aroma. The OAVi for each volatile compound was calculated as OAVi = Ci/Ti; the concentration of compound I was denoted by Ci (µg·kg^−1^), and Ti was the threshold for compound i (µg·kg^−1^).

### 4.6. Determination of All-Element Content in Fresh Tea Leaves

Nitrogen, phosphorus, and potassium elements in the collected tea fresh leaves were determined with reference to the method of NY/T 2017–2011. Following this method, weigh 1 g–5 g (accurate to 0.0001 g) of fresh sample in a 100 mL digestion tube. Add 5 mL of sulfuric acid into the digestion tube, shake well, add hydrogen peroxide two times, 2 mL each time, shake well, cover with a small funnel, and wait for the end of the intense reaction, put it on the decoction furnace to heat and decoct, so that the solids disappeared to become a solution, and wait for the sulfuric acid to emit white smoke, and when the solution became brown, stop heating. After a little cooling, add 10 drops of hydrogen peroxide, continue to heat, and cook for about 5 min; then add 10 drops of hydrogen peroxide to cook, and so on until the solution is clear, and then continue to heat for 5 min. Remove and cool; use water to transfer all the digested solution to a 100 mL volumetric flask, fixed capacity, filtered with filter paper or left to clarify the solution to be used to determine N, P, and K. The solution was used for the determination of N, P, and K. Kjeldahl method, vanadium-molybdenum yellow absorbance photometry, and flame atomic absorption spectrophotometry were used for the determination of N, P, and K content.

Then, accurately weigh 0.50000 g (±0.00005) of tea samples, add 50% nitric acid 10 mL, add and leave to exhaust for more than 8 h, cook at 100 °C until nearly dry, then add 10 mL of 50% nitric acid, and continue to heat until it is clear, and then determine the content of other elements using inductively coupled plasma mass spectrometry (ICP-MS) (Table S3). There were 3 replicates for each sample.

### 4.7. Statistical Analysis

SPSS 27 (SPSS Inc., Chicago, IL, USA) was used to analyze the data results statistically. Differences in the effects of the flavor compounds, aroma compounds, and elements of different Dancong ancient tea gardens were analyzed using one-way ANOVA (One-way ANOVA) and the Tukey HSD statistical method, with *p* < 0.05 considered a significant difference. Statistical analyses and visualization of non-volatile and volatile compounds were performed using GraphPad Prism 9.5.0 software (GraphPad Software, Inc., La Jolla, CA, USA), and the contents are presented as mean ± standard deviation (SD). Principal component analysis (PCA), partial least squares discriminant analysis (PLS-DA), and projected variable importance (VIP) analyses were performed using SIMCA (Version 14.1, Umetrics, Umea, Sweden) software. Heat maps were produced using Tbtools II (Version 2.080, Guangzhou, China) software.

## 5. Conclusions

In this study, four different altitude Dancong ancient tea gardens in Fenghuang Tea District, Chaozhou City, Guangdong Province, were used as the research objects to establish the correlation model of key non-volatile substances, key volatile substances and fresh leaf elements affecting the difference of fresh leaves in different altitude Dancong ancient tea gardens. After statistical analysis, the different contents of EGC, EGCG, and caffeine may have contributed to the differences in the quality of the non-volatile dimensions of the tea leaves from different ancient tea gardens, with the low-altitude tea garden ZMF having a more bitter and astringent taste than the other high-altitude tea gardens due to it containing the highest content of caffeine (5.21%) and a high astringency index (0.82). These two flavor composition indicators had relatively strong positive correlations (0.74 and 0.48) with fresh leaf N content, which may be related to the higher content of N in the fresh leaves of this tea garden. The OAV values were utilized to identify the possible significant contribution of linalool, 4-hexen-1-yl acetate, geraniol, methyl salicylate, *trans*-2-hexenal and decanal to the aroma quality of the tea leaves of the four different ancient tea gardens. The total concentration of volatiles compounds of the low altitude ZMF tea garden was the highest (552.65 μg/kg). Among them, the linalool content of fresh leaves of ZMF and BX of the highest altitude tea gardens at low altitude was significantly higher than that of other tea garden, which, combined with the correlation analysis, might be due to the fact that both tea gardens had lower content of Al element in fresh leaves than the other tea gardens, which reduced the effect on linalool synthesis. In addition, the lower content of Si and Hf in the fresh leaves of the ZMF tea garden also contributed to the higher content of green aroma volatile compounds in the fresh leaves of its tea garden than that of other high-altitude tea gardens. The results of the study provide guidance for the management and resource utilization of ancient tea gardens as well as fertilization strategies in tea cultivation and production, and highlight the outstanding characteristics of ancient Dancong tea gardens fresh leaves.

## Figures and Tables

**Table 1:** Astringency index of fresh leaves from different Dancong ancient tea gardens.

	EGCG	GCG	ECG	Total
ZMF	0.65	0.02	0.14	0.82
BX	0.61	0.02	0.13	0.76
HSK	0.60	0.02	0.13	0.75
DAF	0.55	0.04	0.14	0.70

**Table 2:** Identification of six volatile compounds with VIP > 1 in fresh leaves of four different Dancong ancient tea gardens.

Var. No.	Volatile Compounds	Odor	VIP ^1^	OT ^2^ (μg/kg)	OAVs			
					BX	DAF	HSK	ZMF
var_5	Linalool	Floral	2.73	0.22 ^a^	554.45	184.14	359.21	687.78
var_1	*trans*-2-hexenal	Green	2.44	17 ^b^	4.56	4.01	2.67	6.00
var_29	4-hexen-1-yl acetate	Green	2.31	100 ^b^	0.32	0.61	0.87	1.25
var_13	Geraniol	Floral	2.20	1.1 ^c^	115.72	58.47	29.43	55.54
var_10	Methyl salicylate	Green	1.96	40 ^a^	0.73	0.18	0.86	1.48
var_11	Decanal	Citrus	1.29	0.3 ^d^	28.11	12.44	33.74	14.38

^1^ VIP, variable importance in projection; ^2^ OT, odor threshold value reported, indicated as ^a–d^ [24,25,26,27].

**Table 3:** Geographical distribution of different Dancong ancient tea gardens.

Location	ID	Longitude (°)	Latitude (°)	Altitude (m)
Zimao	ZMF	116.68° E	23.95° N	578.13
Baixiang	BX	116.65° E	23.96° N	1131.63
Xialiao	HSK	116.65° E	23.96° N	1066.77
Da′an	DAF	116.64° E	23.97° N	965.91

## Data Availability

The original contributions presented in the study are included in the article/Supplementary Materials, further inquiries can be directed to the corresponding authors.

## Supplementary Materials

The following supporting information can be downloaded at: https://www.mdpi.com/article/10.3390/plants14091339/s1, Table S1: All elemental contents of fresh leaves in different ancient tea gardens; Table S2 Parameters of the calibration curves for the components; Table S3 Operating Conditions of Inductively Coupled Plasma Mass Spectrometer; Table S4 Types of Volatile Compound Aromas and Reference Literature; Figure S1: Multivariate statistical analysis of elemental contents identified in fresh leaves of four different Dancong ancient tea gardens. References [49,50,51,52,53,54,55,56,57,58] are citied in the Supplementary Materials.
