# Supplementary material for: Impact of Fresh Leaf Elements on Flavor Components and Aroma Quality in Ancient Dancong Tea Gardens Across Varying Altitudes
Source: Plants (Basel). 2025 Apr 29;14(9):1339. doi: 10.3390/plants14091339 (PMC12073577; doi:10.3390/plants14091339)
Supplement: Supplementary file 1 [file plants-14-01339-s001.zip › plants-3572959-supplementary.pdf]

Element	ZMF	BX	HSK	DAF
N	47848.89±823.12 a	43368.89±5488.00 a	39075.56±4370.20 a	43742.22±2856.13 a
P	3640.17±105.68 a	3749.82±82.53 a	3626.36±30.90 a	3514.13±14.52 a
K	11946.67±232.47 a	12906.67±192.30 a	13733.33±707.04 a	12560.00±366.61 a
Ca	1769.46±148.92 a	1348.92±189.78 a	1844.39±217.22 a	2415.45±359.26 a
Mg	1781.58±86.20 a	1801.52±103.21 a	1920.42±135.63 a	2289.17±239.14 a
Fe	211.78±122.58 a	92.90±5.83 a	115.64±13.35 a	135.27±10.29 a
Al	156.50±13.52 b	173.70±18.60 b	581.63±160.93 a	669.99±25.71 a
Mn	259.94±19.87 a	263.23±31.53 a	316.13±76.55 a	333.17±53.47 a
Na	172.31±17.19 a	159.51±22.95 a	587.02±296.64 a	628.34±163.18 a
Si	61.05±5.08 b	78.79±1.22 b	170.37±59.69 ab	170.20±8.67 a
Zn	39.01±3.35 a	39.84±5.63 a	31.54±3.99 a	34.75±3.70 a
B	16.93±1.31 a	16.74±0.61 a	15.35±0.87 a	17.81±5.73 a
Ba	23.84±4.99 a	14.20±1.12 a	24.43±6.26 a	19.03±3.94 a
Li	21.79±4.26 a	18.73±2.10 a	17.95±1.32 a	25.88±10.17 a
Cu	13.62±2.08 a	12.65±1.55 a	12.14±1.54 a	14.78±1.20 a
Sr	24.23±11.04 a	14.23±0.79 a	30.23±11.15 a	15.94±6.15 a
Bi	0.25±0.04 b	0.88±0.15 a	0.11±0.01 b	0.75±0.14 a
Co	0.55±0.38 a	0.17±0.02 a	0.29±0.05 a	0.18±0.07 a
Mo	0.49±0.21 a	0 ns	0.66±0.27 a	0.57±0.03 a
Ti	2.06±0.83 a	3.72±2.12 a	3.47±0.89 a	1.97±0.11 a
Zr	1.00±0.16 ab	0.32±0.09 b	2.52±0.64 a	2.20±0.42 a
Hf	0.10±0.01 b	0.30±0.03 ab	0.40±0.08 a	0.37±0.08 ab
V	0.11±0.04 a	0.31±0.12 a	0.24±0.07 a	0.09±0.01 a
Cr	8.15±0.66 a	8.07±0.33 a	2.43±0.38 b	3.41±0.18 b
As	0.48±0.19 a	0.77±0.29 a	0.38±0.14 a	0.79±0.12 a
Sn	0 ns	0.44±0.11 a	0.27±0.10 a	0.11±0.06 a
Ni	3.41±0.58 a	5.40±0.71 a	5.03±0.60 a	4.12±0.16 a

Table S1 All elemental contents of fresh leaves in different Dancong ancient tea gardens. Letters followed the content represent significant levels ($p < 0.05$), and letters indicate differences in elemental contents in fresh leaves of different Dancong ancient tea gardens.

Name	Equation	R^2
Caffein	Y = 2.44e+007 X + 2.04e+004	0.999807
Theanine	Y = 8.61e+006 X + 1.71e+005	0.99962
GA	Y = 3.25e+007 X + 1.19e+005	0.999937
GC	Y = 2.18e+006 X + 4.29e+003	0.999956
EGC	Y = 2.17e+006 X + 3.77e+004	0.998972
C	Y = 7.30e+006 X + 5.07e+004	0.99991
EC	Y = 8.02e+006 X + 1.92e+005	0.996947
EGCG	Y = 1.59e+007 X + 1.17e+005	0.999966
GCG	Y = 1.58e+007 X + 3.54e+005	0.997493
ECG	Y = 1.91e+007 X + 8.41e+005	0.999046

Table S2 Parameters of the calibration curves for the components.

Name	Equation	Name	Equation
RF Power	1600 W	Nebulizer	0.67 L/min
Plasma Gas Flow Rate	18 L/min	Sampler Cone/Skimmer Cone	Nickel cone
Omega Deflection Voltage	-130 V	Omega Lens Voltage	11.4 V
Carrier Gas Flow Rate	0.9 L/min	Sampling Depth	8 mm
Auxiliary Gas Flow Rate	1 L/min	Sampling Mode	Peak Hopping
Helium Gas Flow Rate	3.8 L/min	Collision Cell Mode	KED
Nebulizer Chamber Temperature	2 ℃	Points per Peak	1-3
Pump Speed	0.1 rps	Replicates	3

Table S3 Operating Conditions of Inductively Coupled Plasma Mass Spectrometer.

	Odor Type	Reference
trans-2-hexenal	Green	[1]
β-myrcene	Woody	[2]
β-ocimene	Citrus	[2]
trans-Linalool oxide (furanoid)	Floral	[3]
Linalool	Floral	[4]
Alloocimene	Floral	[3]
4-Terpineol	Woody	[4]
α-Terpineol	Floral	[5]
Methyl salicylate	Green	[4]
Decanal	Citrus	[6]
cis-Nerol	Citrus	[7]
Geraniol	Floral	[4]
Citral	Citrus	[6]
Copaene	Floral	[8]
cis-3-methyl-2-(2-pentenyl)-2-Cyclopenten-1-one	Floral	[9]
Caryophyllene	Woody	[2]
α-Ionone	Floral	[5]
trans-6,10-dimethyl-5,9-Undecadien-2-one	Green	[10]
cis-β-farnesene	Floral	[2]
trans-β-Ionone	Floral	[2]
(+)-Δ-Cadinene	Woody	[9]
Nerolidol	Floral	[11]
Fitone	Floral	[12]
4-hexen-1-yl acetate	Green	[2]
β-cyclocitral	Fruity	[8]
spiroxide	Woody	[7]

Table S4 Types of Volatile Compound Aromas and Reference Literature. (See the 'Extended reference list' for references)

Extended reference list

1. Wang, H.; Shen, S.; Wang, J.; Jiang, Y.; Li, J.; Yang, Y.; Hua, J.; Yuan, H. Novel Insight into the Effect of Fermentation Time on Quality of Yunnan Congou Black Tea. *LWT* **2022**, *155*, 112939, doi:10.1016/j.lwt.2021.112939.

2. Guo, X.; Ho, C.-T.; Wan, X.; Zhu, H.; Liu, Q.; Wen, Z. Changes of Volatile Compounds and Odor Profiles in Wuyi Rock Tea during Processing. *Food Chemistry* **2021**, *341*, 128230, doi:10.1016/j.foodchem.2020.128230.

3. Guo, X.; Schwab, W.; Ho, C.-T.; Song, C.; Wan, X. Characterization of the Aroma Profiles of Oolong Tea Made from Three Tea Cultivars by Both GC–MS and GC-IMS. *Food Chemistry* **2022**, *376*, 131933, doi:10.1016/j.foodchem.2021.131933.

4. Wang, M.; Li, J.; Liu, X.; Liu, C.; Qian, J.; Yang, J.; Zhou, X.; Jia, Y.; Tang, J.; Zeng, L. Characterization of Key Odorants in Lingtou Dancong Oolong Tea and Their Differences

Induced by Environmental Conditions from Different Altitudes. *Metabolites* **2022**, *12*, 1063, doi:10.3390/metabo12111063.

5. Zhai, X.; Zhang, L.; Granvogl, M.; Ho, C.; Wan, X. Flavor of Tea (*Camellia Sinensis*): A Review on Odorants and Analytical Techniques. *Comp Rev Food Sci Food Safe* **2022**, *21*, 3867–3909, doi:10.1111/1541-4337.12999.
6. Fang, X.; Liu, Y.; Xiao, J.; Ma, C.; Huang, Y. GC–MS and LC-MS/MS Metabolomics Revealed Dynamic Changes of Volatile and Non-Volatile Compounds during Withering Process of Black Tea. *Food Chemistry* **2023**, *410*, 135396, doi:10.1016/j.foodchem.2023.135396.
7. Guo, X.; Ho, C.-T.; Schwab, W.; Wan, X. Aroma Profiles of Green Tea Made with Fresh Tea Leaves Plucked in Summer. *Food Chemistry* **2021**, *363*, 130328, doi:10.1016/j.foodchem.2021.130328.
8. Wang, M.-Q.; Ma, W.-J.; Shi, J.; Zhu, Y.; Lin, Z.; Lv, H.-P. Characterization of the Key Aroma Compounds in Longjing Tea Using Stir Bar Sorptive Extraction (SBSE) Combined with Gas Chromatography-Mass Spectrometry (GC–MS), Gas Chromatography-Olfactometry (GC-O), Odor Activity Value (OAV), and Aroma Recombination. *Food Research International* **2020**, *130*, 108908, doi:10.1016/j.foodres.2019.108908.
9. Guo, X.; Ho, C.-T.; Schwab, W.; Wan, X. Effect of the Roasting Degree on Flavor Quality of Large-Leaf Yellow Tea. *Food Chemistry* **2021**, *347*, 129016, doi:10.1016/j.foodchem.2021.129016.
10. Zhang, W.; Cao, J.; Li, Z.; Li, Q.; Lai, X.; Sun, L.; Chen, R.; Wen, S.; Sun, S.; Lai, Z. HS-SPME and GC/MS Volatile Component Analysis of Yinghong No. 9 Dark Tea during the Pile Fermentation Process. *Food Chemistry* **2021**, *357*, 129654, doi:10.1016/j.foodchem.2021.129654.
11. Hao, Z.; Feng, J.; Chen, Q.; Lin, H.; Zhou, X.; Zhuang, J.; Wang, J.; Tan, Y.; Sun, Z.; Wang, Y.; et al. Comparative Volatiles Profiling in Milk-Flavored White Tea and Traditional White Tea Shoumei via HS-SPME-GC-TOFMS and OAV Analyses. *Food Chemistry: X* **2023**, *18*, 100710, doi:10.1016/j.fochx.2023.100710.
12. Xiao, L.; Yang, C.; Zhang, X.; Wang, Y.; Li, Z.; Chen, Y.; Liu, Z.; Zhu, M.; Xiao, Y. Effects of Solid-State Fermentation with Bacillus Subtilis LK-1 on the Volatile Profile, Catechins Composition and Antioxidant Activity of Dark Teas. *Food Chemistry: X* **2023**, *19*, 100811, doi:10.1016/j.fochx.2023.100811.

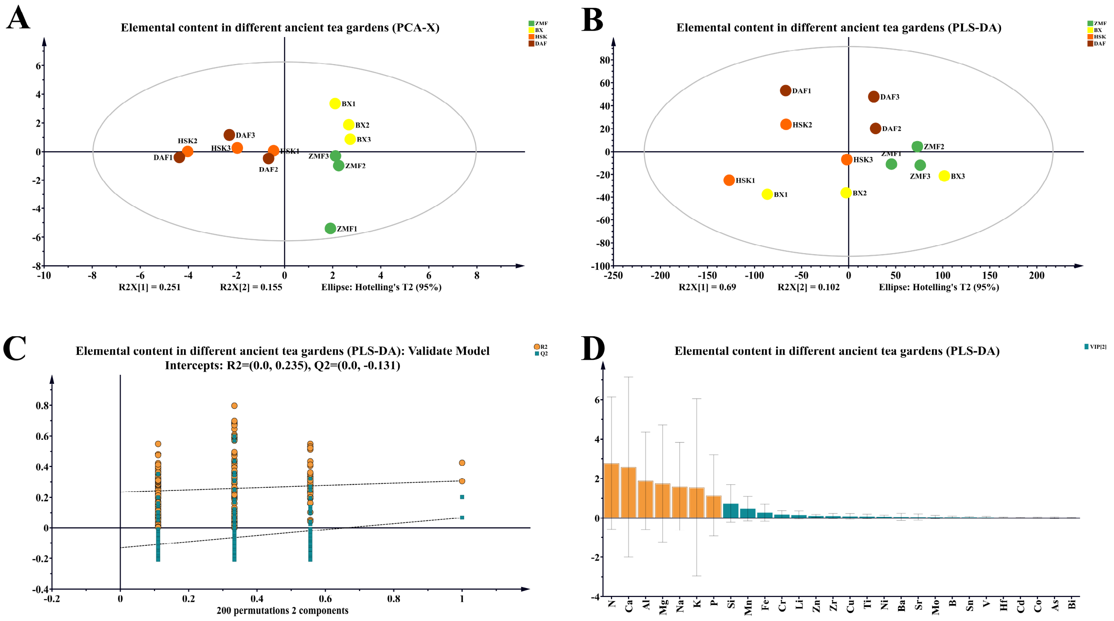

Figure S1 Multivariate statistical analysis of elemental contents identified in fresh leaves of four different Dancong ancient tea gardens. (A) PCA plots of elemental contents in fresh leaves of different Dancong ancient tea gardens; (B) PLS-DA score plots of elemental contents in fresh leaves of different Dancong ancient tea gardens; (C) VIP scoring plots: orange color bars indicate volatile compounds with VIP > 1; green color indicates volatile compounds with VIP < 1. (D) Cross-validation results: 200 comparisons of the cross-validated model, the intercept of the Q2 replica line is less than 0, indicating that the PLS-DA discriminant model is not overfitted and the model is relatively reliable.
